# IgG1 Is Required for Optimal Protection after Immunization with the Purified Porin OmpD from *Salmonella* Typhimurium

**DOI:** 10.4049/jimmunol.1700952

**Published:** 2017-11-10

**Authors:** Yang Zhang, Coral Dominguez-Medina, Nicola J. Cumley, Jennifer N. Heath, Sarah J. Essex, Saeeda Bobat, Anna Schager, Margaret Goodall, Sven Kracker, Christopher D. Buckley, Robin C. May, Robert A. Kingsley, Calman A. MacLennan, Constantino López-Macías, Adam F. Cunningham, Kai-Michael Toellner

**Affiliations:** * Institute of Immunology and Immunotherapy, University of Birmingham, Birmingham B15 2TT, United Kingdom;; † School of Biosciences, University of Birmingham, Birmingham B15 2TT, United Kingdom;; ‡ School of Cancer Sciences, University of Birmingham, Birmingham B15 2TT, United Kingdom;; § Deutsches Rheuma-Forschungszentrum Berlin, Berlin 10117, Germany;; ¶ Wellcome Trust Sanger Institute, Cambridge CB10 1SA, United Kingdom;; ‖ Medical Research Unit on Immunochemistry, Specialties Hospital, National Medical Centre Siglo XXI, Mexican Social Security Institute, 06720 México, DF, Mexico; and; # Institute of Microbiology and Infection, University of Birmingham, Birmingham B15 2TT, United Kingdom

## Abstract

In mice, the IgG subclass induced after Ag encounter can reflect the nature of the Ag. Th2 Ags such as alum-precipitated proteins and helminths induce IgG1, whereas Th1 Ags, such as *Salmonella* Typhimurium, predominantly induce IgG2a. The contribution of different IgG isotypes to protection against bacteria such as *S.* Typhimurium is unclear, although as IgG2a is induced by natural infection, it is assumed this isotype is important. Previously, we have shown that purified *S.* Typhimurium porins including outer membrane protein OmpD, which induce both IgG1 and IgG2a in mice, provide protection to *S.* Typhimurium infection via Ab. In this study we report the unexpected finding that mice lacking IgG1, but not IgG2a, are substantially less protected after porin immunization than wild-type controls. IgG1-deficient mice produce more porin-specific IgG2a, resulting in total IgG levels that are similar to wild-type mice. The decreased protection in IgG1-deficient mice correlates with less efficient bacterial opsonization and uptake by macrophages, and this reflects the low binding of outer membrane protein OmpD–specific IgG2a to the bacterial surface. Thus, the Th2-associated isotype IgG1 can play a role in protection against Th1-associated organisms such as *S.* Typhimurium. Therefore, individual IgG subclasses to a single Ag can provide different levels of protection and the IgG isotype induced may need to be a consideration when designing vaccines and immunization strategies.

## Introduction

Pathogen-specific Ab is the critical factor conferring immunity in response to most vaccines. IgG-switched Ab is often measured as the main mediator of immunity and is often sufficient to confer protection after passive vaccination. IgG can work through neutralization, through binding complement, and through the activation of a range of Fcγ receptors. These activities show some variability depending upon which isotype of IgG is being examined. Different IgG isotypes of man and mouse are not evolutionary homologs, but there are similarities in some features such as a similar diversity in flexibility or different capacities to interact with activating and inhibitory Fc receptors and complement (1). Therefore, an IgG response involving multiple isotypes can lead to a complex network of modulatory effects mediated by a range of different IgG subclasses.

In mice, the direction of IgG switching can reflect the polarization of the Th cell response, which is determined by the nature of the immunizing Ag (2). Immunization with soluble flagellin, most pure proteins precipitated on alum, or helminths such as *Nippostrongylus brasiliensis* induces a strong Th type 2 response with class switching to IgG1 and little detectable IgG2a (3, 4). The induction of IgG1, in vitro, is strongly dependent upon IL-4, but in vivo this is not essential (5). Murine IgG1 has a modest capacity to bind C1q and because it binds weakly to FcγRIIB and FcγRIII, it is not associated with promoting strong activation of immune responses in host immune cells (6, 7). Therefore, IgG1 is not considered to play significant roles in protection against intracellular bacterial infections, although IgG1 promotes protection against other pathogens, e.g., *Cryptococcus neoformans* (8).

Infection with live Gram-negative bacteria such as *Salmonella enterica* serovar Typhimurium induces Th1 immunity with less IgG1 and dominant switching to IgG2a, associated with the transcription factor T-Bet (9). Because Ab induced after natural infection with *S.* Typhimurium can protect, it seems reasonable to assume that Th1-associated IgG isotypes, such as IgG2a, play a key role in this protection.

Immunization with the porin protein outer membrane protein OmpD (OmpD) from *S.* Typhimurium can provide Ab-mediated protection against infection, with IgG contributing the majority of this effect (10). Immunization with purified porins confers immunity to *S.* Typhimurium infection and induces both IgG1 and IgG2a (11). It is not clear whether different isotypes contribute equally to protection. In this study we have tested the requirement for IgG1 for protection against *S.* Typhimurium infection after immunization with OmpD. Unexpectedly, we identify a key role for IgG1 in mediating vaccine-induced protection from infection.

## Materials and Methods

### Mice

Sex-matched 6–12 wk old C57BL/6 mice were obtained from Harlan Laboratories (Bicester, U.K.). hMt mice with a targeted deletion of the splice site of IgG1 H chain germline transcripts are unable to class switch to IgG1-deficient hMt mice (IgG1ko) (12), T-bet–deficient mice (T-betKO) (13), and mice in which the Cre-coding sequence was inserted into the 3′ region of the Cγ1 locus (Cγ1-Cre) (14) were bred in house. Animal studies were performed with the approval of local ethical committees and under Home Office license.

### Bacterial strains, immunizations, infections, and opsonization of bacteria

*S.* Typhimurium SL3261 is an attenuated mutant of the laboratory strain SL1344 (15). *S.* Typhimurium SL3261 expressing GFP was generated by inserting the eGFP gene into the pettac plasmid, which has a modified tac promoter to enable constitutive gene expression, as described previously (16). *S.* Typhimurium SL1344 deficient for OmpF and OmpC (ompC::aph ompF::cat) was used as the bacterial source of OmpD (10); *S.* Typhimurium D23580 is a virulent, representative isolate of an invasive *S.* Typhimurium strain from sub-Saharan Africa (17). *S.* Typhimurium D23580 deficient for *wbaP* are unable to make LPS containing *O*-Ag. Genetically altered strains were generated by P22 transduction using standard methods (18).

OmpD preparations from *S.* Typhimurium were made by 2% (v/v) Triton X-100 extraction (19) and repeated extraction with SDS and separation by fast protein liquid chromatography on a Sephacryl S-200 column before dialysis against PBS/0.1% (w/v) SDS (10, 20). By SDS-PAGE with detection of protein by Gelcode Blue, OmpD is a single band and the limulus assay estimates endotoxin contamination to be typically one endotoxin unit per 30 μg of OmpD. Mice were primed with 20 μg OmpD in PBS i.p. Then 5 wk later, mice were infected with 5 × 10^5^ *S.* Typhimurium i.p. In some experiments mice were boosted with 20 μg OmpD i.p., followed by infection 14 d later. To obtain OmpD-specific sera, C57BL/6 mice were immunized with 20 μg of OmpD via i.p., boosted on days 14 and 28, and then sacrificed at day 35. Infections with opsonized bacteria were performed as described previously (21), opsonizing with sera from wild-type (WT) and IgG1ko mice that had been primed and boosted with OmpD and infected with *S.* Typhimurium for 5 d. Sera from *S.* Typhimurium–immune mice were matched to contain similar concentrations of OmpD-specific IgG. Sera were heat inactivated at 56°C for 30 min. Then bacteria (2.5 × 10^6^ per ml) and sera (diluted 1:100) were mixed for 30 min and opsonized bacteria were injected into mice i.p. Viability and bacterial aggregation of opsonized bacteria was confirmed to be unaffected by opsonization. Adoptive transfer of serum was performed by injecting 120 μl of sera i.p. into mice 24 h before infection. These sera were from WT or IgG1ko mice immunized twice with OmpD, and had similar levels of total anti-OmpD IgG Ab titers. Tissues were harvested postinfection and bacterial burdens were determined by serial dilution culture of homogenized tissue.

### Immunohistology

Immunohistology was performed on frozen sections as described previously (22). Briefly, acetone-fixed frozen spleen sections (6 μm) were stained using rat anti-mouse IgD (BD Biosciences, Oxford, U.K.), sheep anti-mouse IgD (Abcam, Cambridge, U.K.), rat anti-mouse IgG1, IgG3, IgG2a/c (AbD Serotec, Oxford, U.K.), or 4-hydroxy-nitrophenyl (NP) conjugated to sheep Ig. Secondary Abs (rabbit anti-rat Ig or donkey anti-sheep Ig) conjugated to biotin or HRP were applied. The biotinylated secondary Abs were detected using biotin-conjugated StreptABComplex-alkaline phosphatase complex (Dako, Ely, U.K.). In the final step, color was developed using FastBlue and 3,3′-diaminobenzidine (Sigma-Aldrich). The area of the spleen occupied by germinal centers (GCs) and cells per square millimeter was calculated using a point-counting technique as described previously (22, 23).

### ELISA

ELISAs were performed as previously described to detect serum Abs to NP, *Salmonella* bacteria, or OmpD (19). Plates were coated with Ag at 5 μg/ml overnight. NP_15_-BSA–coupled plates were used to detect NP-specific Ab; NP_2_-BSA–coupled plates were used to measure the high-affinity Ab fraction. Relative affinity was calculated by dividing relative Ab concentration from NP_2_-BSA–coupled plates by the concentration derived from NP_15_-BSA–coupled plates. Alkaline-phosphatase–labeled secondary Abs to IgG (cat: 1030-04), IgG1 (cat: 1070-04), and IgG2a/c (cat: 1080-04), IgG3 (cat: 1079-04), and IgM (1020-04) were purchased from Southern Biotech. The absorbance at OD_405 nm_ was determined using an Emax microplate spectrophotometer (Molecular Devices, Biberach an der Riss, Germany). Relative reciprocal titers were calculated by measuring the dilution at which the serum reached a defined OD_405 nm_.

### In vitro macrophage uptake assay

The semiadherent macrophage-like cell line J774 (24) was used in this assay, which was performed as described previously (25). *S.* Typhimurium–GFP bacteria were opsonized with WT or IgG1ko sera as describe above. Opsonized bacteria were mixed with macrophages in a 5:1 ratio, grown on glass coverslips in serum-free RPMI 1640 medium, and incubated at 37°C for 1 h. Nonadherent bacteria were washed off with PBS 1 h later, and fixed in 4% paraformaldehyde. The coverslips were mounted on microscope slides and visualized on a Zeiss LSM 510 Meta confocal with 100× lens. Fluorescence photomicrographs were taken with a Leica DM6000 with filters suitable for GFP detection. Macrophage cell bodies were segmented using ImageJ/Fiji image analysis software (26) by thresholding the autofluorescent signal obtained at 600 ± 20 nm after 546 nm excitation. The percentage of macrophage area covered by GFP was calculated from >1000 cells per sample.

### Statistics

Statistical analysis was carried out using Prism 6. Groups were compared using unpaired *t* test or Mann–Whitney *U* test as indicated in the figure legends. Correlations were tested using the Spearman rho test. Statistics throughout were carried out by pooling data from all independent experiments. The *p* values are indicated throughout with *p < 0.05, **p < 0.01, ***p < 0.001, and ****p < 0.0001.

## Results

### IgG1-deficient mice respond normally to alum-precipitated proteins or live *S.* Typhimurium

**FIGURE 1.**
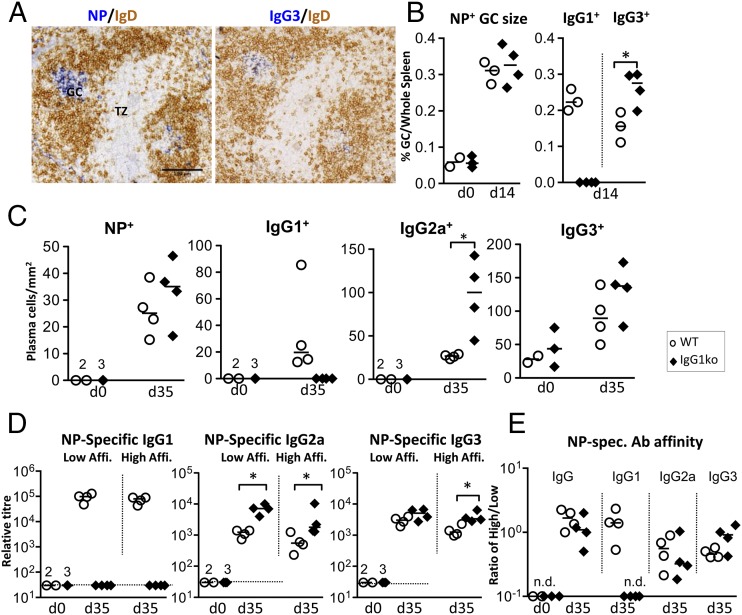
IgG1 deficiency does not affect the extent and affinity of the responses after immunization with T dependent Ag. (**A**) Two adjacent spleen sections from IgG1ko mice showing NP-specific GCs with B cells switched to IgG3; 2 wk after immunization with NP-CGG. Scale bar, 100 μm. (**B**) Size and extent of Ag-specific GCs and GCs containing IgG1- or IgG3-switched B cells in spleens from WT (○) or IgG1ko (♦) mice. (**C**) Numbers of NP-specific plasma cells or plasma cells switched to different IgG isotypes in splenic red pulp. (**D**) Relative amount of total or high-affinity NP-specific Ab of different IgG subclasses in mice before or 5 wk after immunization with NP-CGG. (**E**) Relative affinity of Ag-specific sera shown in (D). Broken horizontal line: detection level. Each symbol represents one animal. Statistical analysis of cell numbers was carried out using a two-tailed *t* test, whereas analysis of Ab titers was carried out using a one-tailed Mann–Whitney *U* test. Experiments were repeated twice with groups containing four mice per group. *p < 0.05. n.d., not detectable; TZ, T cell zone.

Immunization with purified alum-precipitated proteins preferentially induces IgG1 (2). To test how an inability to produce IgG1 affects the Ab response to protein, IgG1ko and WT mice were immunized with alum-precipitated NP coupled to chicken γ globulin (CGG). Then 14 d postimmunization IgG1ko spleens contained NP-specific GCs of comparable number, size, and morphology as WT spleens (Fig. 1A, 1B). B cells in these GCs express IgG subclasses other than IgG1, including IgG3 (Fig. 1A, 1B). Then 5 wk postimmunization, IgG1ko spleens contained similar numbers of NP-specific plasma cells, but increased numbers of plasma cells expressing IgG2a (Fig. 1C). Whereas serum levels of NP-specific IgM were similar (data not shown), Ag-specific IgG3 and IgG2a levels were increased in the absence of IgG1 (Fig. 1D). No significant changes in the amounts of Ag-specific IgE produced and no change in the overall affinity of IgG compared with WT mice were observed (Fig. 1E). Nor could we detect changes in the extent of B and T cell reactivation or Th2-type cytokine production after secondary challenge with the same Ag (data not shown). This shows that affinity maturation and GC differentiation function normally in IgG1ko mice, with class-switching to other isotypes compensating for the loss of IgG1.

**FIGURE 2.**
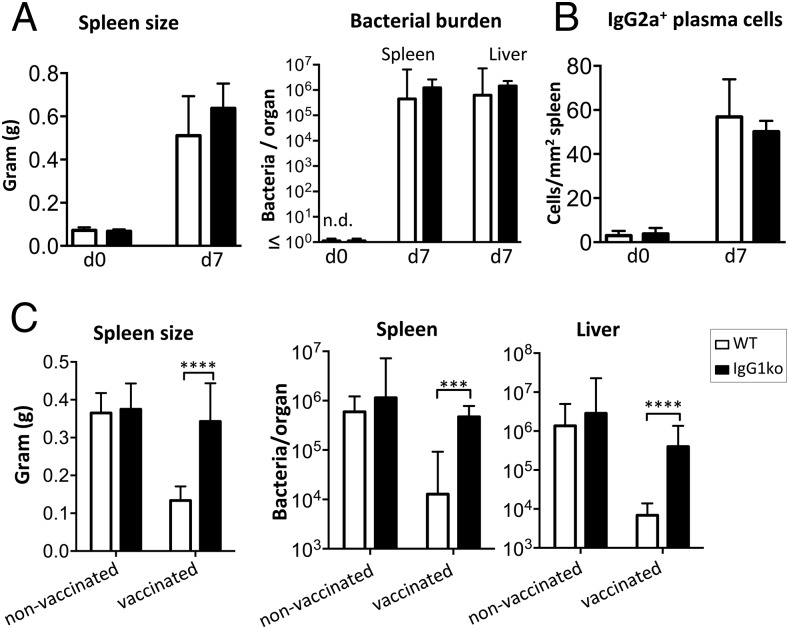
Effect of IgG1 deficiency on *S.* Typhimurium infection. WT (white bars) and IgG1ko (black bars) mice were infected i.p. with 5 × 10^5^ *S.* Typhimurium. (**A**) Spleen mass of naive IgG1ko and WT mice before and 7 d after primary *S.* Typhimurium infection, and bacterial burden in spleen and liver. (**B**) IgG2a-switched plasma cells in spleens of naive mice before and 7 d postinfection. (**C**) Effect of *S.* Typhimurium infection on naive or OmpD-vaccinated WT and IgG1ko mice. Then 35 d after vaccination with 20 μg OmpD mice were challenged with of 5 × 10^5^ *S.* Typhimurium. Responses assessed 5 d postinfection. Spleen mass and bacterial CFU in spleen and liver. Linear scale bar charts show arithmetic mean with SD. Logarithmic bar charts show geometric mean with 95% confidence limits. Data analyzed by two-tailed *t* test. Data are representative of two to three independent experiments with groups containing at least four mice per group. ***p < 0.001, ****p < 0.0001.

We have previously described that the primary Ab response postinfection with live *S.* Typhimurium bacteria induces few splenic IgG1-switched plasma cells and minimal levels of Ag-specific IgG1 to outer membrane Ags (19, 27). This means it differs markedly from the responses to protein Ags in alum or after vaccination with purified porin proteins like OmpD, where Ag-specific responses are readily detectable (2, 27). To confirm that IgG1 does not play a major role during primary infection with *S.* Typhimurium, we infected IgG1ko mice with *S.* Typhimurium. IgG1ko mice had similar levels of splenomegaly and bacterial burdens in the first week of infection, showing they control the infection to a comparable level (Fig. 2A). IgG2a-switched plasmablast induction at 7 d postinfection was similar in both groups (Fig. 2B). Thus, although loss of IgG1 results in a distinct IgG isotype profile, the quality of the primary Ab response to immunization with protein as well as primary infection with *S.* Typhimurium is unaltered.

### Protection to *S.* Typhimurium induced after immunization with OmpD requires IgG1

To investigate whether IgG1 aids protection against *S.* Typhimurium infection after immunization, WT and IgG1ko mice were primed with OmpD purified from *S.* Typhimurium, which can provide Ab-dependent protection in WT mice (10). Then, 5 wk after primary immunization mice were challenged with 5 × 10^5^ *S.* Typhimurium and bacterial burdens assessed 5 d later. Surprisingly, immunized IgG1ko mice were less protected than control mice, with spleens substantially larger (Fig. 2C) and bacterial counts in the liver and spleen higher than in WT mice (Fig. 2C). This indicates that IgG1 is important for the control of *S.* Typhimurium infection in OmpD-immunized mice.

**FIGURE 3.**
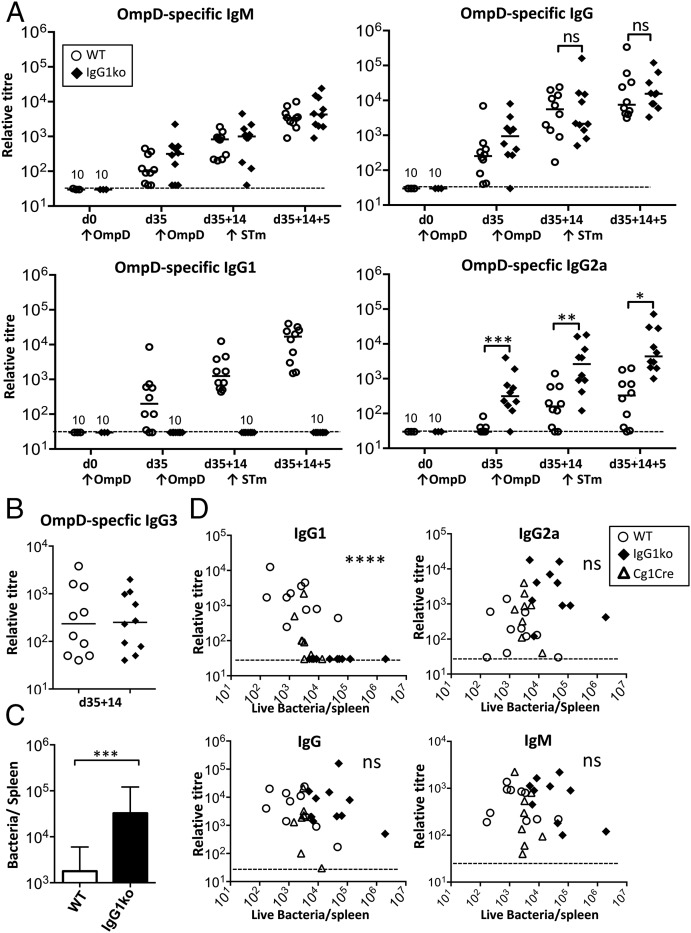
Protection from *S.* Typhimurium infection correlates with IgG1 levels. (**A**) WT (○), or IgG1ko (♦) mice were vaccinated with 20 μg OmpD and boosted at day 35 for 14 d before being infected i.p. with 5 × 10^5^ *S.* Typhimurium. Relative levels of OmpD-specific IgM, IgG, IgG1, and IgG2a in sera assessed by ELISA. (**B**) Relative levels of OmpD-specific IgG3 in sera from mice 14 d after a second immunization with OmpD and prior to infection. Statistical analysis using one-tailed Mann–Whitney *U* test. Broken line: detection level. (**C**) Bacterial numbers in spleens in the same mice 5 d postinfection with *S.* Typhimurium. Statistical analysis was carried using two-tailed Student *t* test from data of two independent experiments. Bars and whiskers indicate geometric mean and 95% confidence limits. (**D**) Correlation of OmpD-specific serum Ab levels of different Ig classes before infection and matched splenic bacterial count 5 d postinfection. WT (○), IgG1ko (♦), and Cg1-Cre (∆) mice vaccinated and infected as in (A). Statistical analysis was performed using the Spearman rho test. Data plotted are from two independent experiments, with five mice in each group. Each symbol corresponds to one animal. *p < 0.05, **p < 0.01, ***p < 0.001, ****p < 0.0001. ns, not significant.

A second immunization with protein Ags should lead to increased levels and higher affinity of OmpD-specific Ab. To test whether a further immunization can compensate for the absence of IgG1, WT and IgG1ko mice were reimmunized with OmpD before challenge with *S.* Typhimurium. OmpD-specific IgM and overall IgG levels were similar between WT and IgG1ko mice throughout this response. Similar to what is seen in responses to NP-CGG, IgG1ko mice compensate for the loss of IgG1 through higher levels of IgG2a (Fig. 3A). OmpD-specific IgG3 titers were similar between both groups (Fig. 3B). Challenge with live *S.* Typhimurium did not lead to a change in the bias toward IgG1 (Fig. 3A). Assessing bacterial numbers in spleen 5 d postinfection showed that prime-boosted IgG1ko mice also had splenic bacterial burdens >20-fold higher than WT mice (Fig. 3C), indicating the additional Ab induced is not sufficient to compensate for the loss of IgG1.

To test protection in animals expressing a wider range of Ag-specific IgG1 and IgG2a titers, we used the same immunization and challenge protocol with Cγ1-Cre mice, which can switch to IgG1 but at much reduced levels (14). We then correlated bacterial burdens against Ab titers. This showed that the level of protection from *S.* Typhimurium infection correlated with OmpD-specific IgG1, but not with other Ig classes (Fig. 3D). Thus, IgG1 is needed for optimal protection after immunization with OmpD and the level of specific IgG1 induced correlates with the level of protection.

### IgG2a is not essential for protection after immunization with OmpD

**FIGURE 4.**
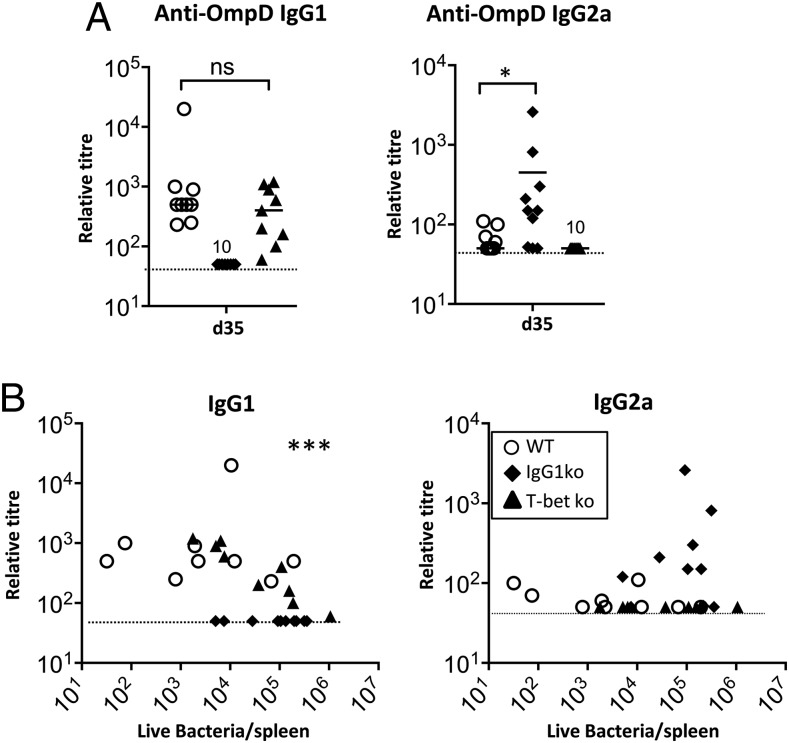
Protection after OmpD immunization is maintained in the absence of IgG2a. WT, IgG1ko, and T-betKO were immunized with OmpD and challenged with *S.* Typhimurium at day 35. (**A**) OmpD-specific IgG1 and IgG2a levels prior to challenge and 5 d postinfection with *S.* Typhimurium. (**B**) Matched OmpD-specific IgG1 and IgG2a serum levels prior to challenge with *S.* Typhimurium and splenic bacterial numbers 5 d after challenge. Statistical analysis performed using the Spearman rho test. Data plotted are from two independent experiments, with groups each containing five mice. Each symbol corresponds to one animal. Broken horizontal line: detection level. *p < 0.05, ***p < 0.001. ns, not significant.

IgG2a is induced after natural infection with *S.* Typhimurium and therefore may contribute to protection. To test this, WT, IgG1ko, and T-betKO mice (which do not produce IgG2a) were immunized with OmpD and challenged with *S.* Typhimurium 35 d later (Fig. 4A). Immunization induced similar IgG1 titers in the WT and T-betKO mice. As expected, IgG2a was not detectable in T-betKO mice (Fig. 4A). Comparing OmpD-specific IgG subclass levels and bacterial burdens showed a clear negative correlation with Ag-specific IgG1, but not IgG2a levels (Fig. 4B). This is consistent with IgG1 having an important role for protection after immunization with OmpD but IgG2a being less important.

### IgG1 promotes bacterial adhesion and uptake by phagocytes

**FIGURE 5.**
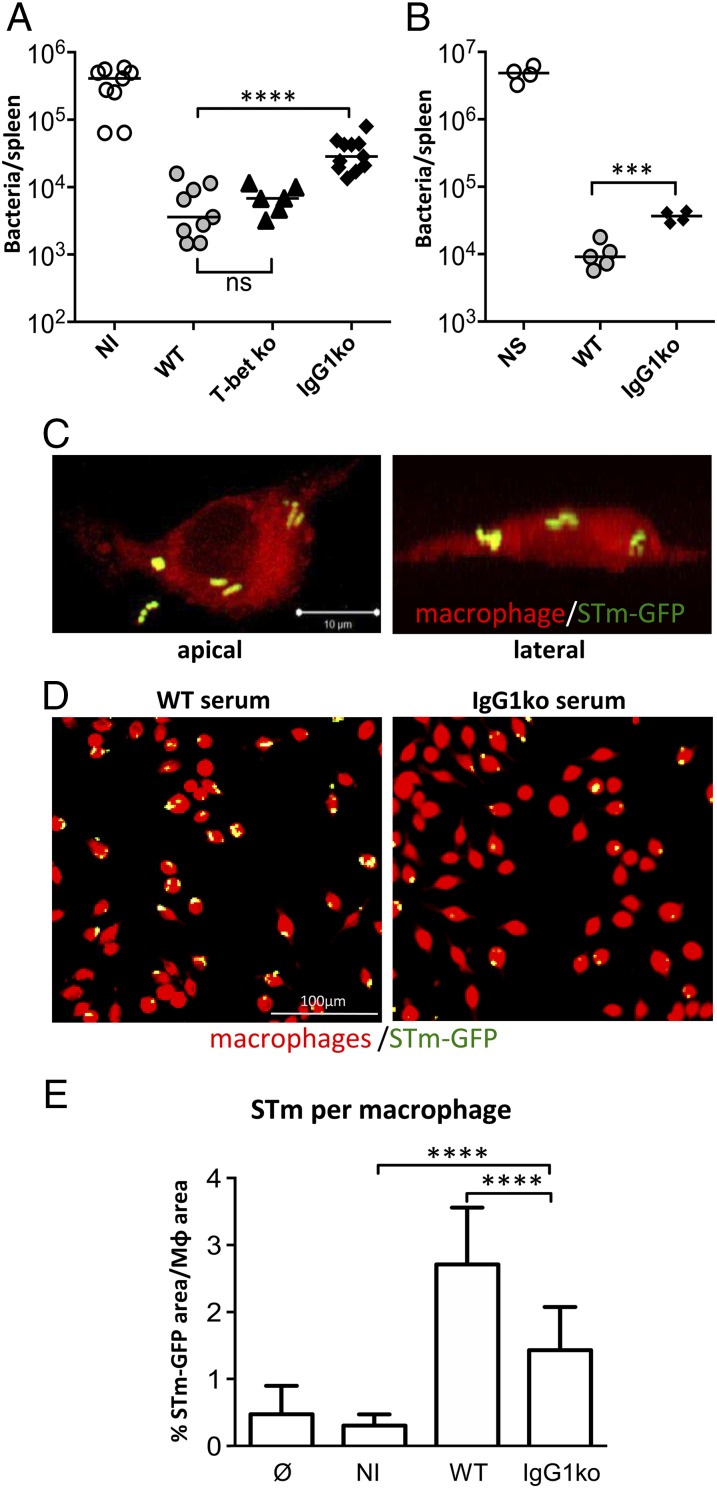
IgG1 promotes the association of bacteria and phagocytes. (**A**) B cell–deficient mice were infected with *S.* Typhimurium opsonized with sera from nonimmune (NI) or OmpD-immunized WT, T-betKO, or IgG1ko mice (matched to contain similar levels of OmpD-specific IgG). Splenic bacterial numbers were quantified 5 d postinfection. Each symbol corresponds to one mouse. Statistical analysis using two-tailed *t* test. (**B**) WT mice were injected with sera from OmpD-immunized WT or IgG1ko mice (matched to contain similar levels of OmpD-specific total IgG) or no sera (NS), and 24 hr later infected with *S.* Typhimurium SL3261. Splenic bacterial numbers were quantified 5 d postinfection. (**C**) Apical and lateral view of a J774 macrophage after 1 h culture with GFP-expressing *S.* Typhimurium opsonized with OmpD-specific sera from a WT mouse. Green, *S.* Typhimurium–GFP; red, autofluorescence. (**D**) Overview fluorescence micrographs of J774 cells incubated with GFP-expressing *S.* Typhimurium opsonized with OmpD-specific WT or IgG1ko sera. (**E**) Quantification of *S.* Typhimurium uptake from macrophages incubated with *S.* Typhimurium not opsonized or incubated with Ag-naive WT serum (NI) or immune WT or IgG1ko serum. Bars show mean and SD derived from >10 microphotographs per sample. In (A) and (B), each group contained at least three mice and are shown from three experiments, except for (B), which was performed once. In (C)–(E), data are representative of three independent experiments, with signal strength calculated from >1000 cells analyzed for each condition. Statistical analysis using two-tailed *t* test, ***p < 0.001, ****p < 0.0001.

We then examined whether the Ab itself from OmpD-immunized mice was responsible for these effects. To do this, sera from WT, IgG1ko, or T-betKO mice (all immunized twice with OmpD) were matched to ensure the OmpD-specific total IgG titers were comparable. Sera were heat treated to inactivate complement. Bacteria were then opsonized with these sera and with control sera from nonimmunized WT mice and mice were infected with these opsonized bacteria. Opsonization with sera from OmpD-immunized WT or T-betKO mice led to an approximate 100-fold lower number of bacteria in spleens than infection with bacteria incubated with nonimmune sera (Fig. 5A). In contrast, bacterial numbers in spleens of mice infected with *S.* Typhimurium opsonized with IgG1-deficient sera were ∼10-fold higher than in mice that received *S.* Typhimurium opsonized with OmpD-specific sera from WT or T-betKO mice (Fig. 5A). Similarly, transfer of OmpD-specific serum from WT or IgG1ko mice into naive recipients 1 d before challenge with *S.* Typhimurium led to reduced protection if IgG1 was absent (Fig. 5B).

In mice, killing of *S.* Typhimurium is strongly reliant upon cell-dependent mechanisms through enhancing bacterial uptake by phagocytes (28, 29). To test whether loss of IgG1 affected bacterial uptake by macrophages, *S.* Typhimurium–expressing GFP were opsonized with sera from naive or OmpD-immunized WT or IgG1ko mice, which were matched to have similar total anti-OmpD–specific IgG titers. Opsonized bacteria were then incubated in vitro with the murine macrophage cell line J774 and attachment and uptake of bacteria by macrophages assessed by confocal microscopy. This showed bacteria in contact with and apparently ingested by macrophages (Fig. 5C). Quantification of the bacteria associated with phagocytes revealed fewer bacteria colocalized with macrophages when IgG1 was absent (Fig. 5D, 5E). This suggests that opsonization of bacteria by IgG1 promotes the binding and uptake of bacteria by phagocytes.

### IgG2a shows poor binding to OmpD in its natural context on the bacterial surface

**FIGURE 6.**
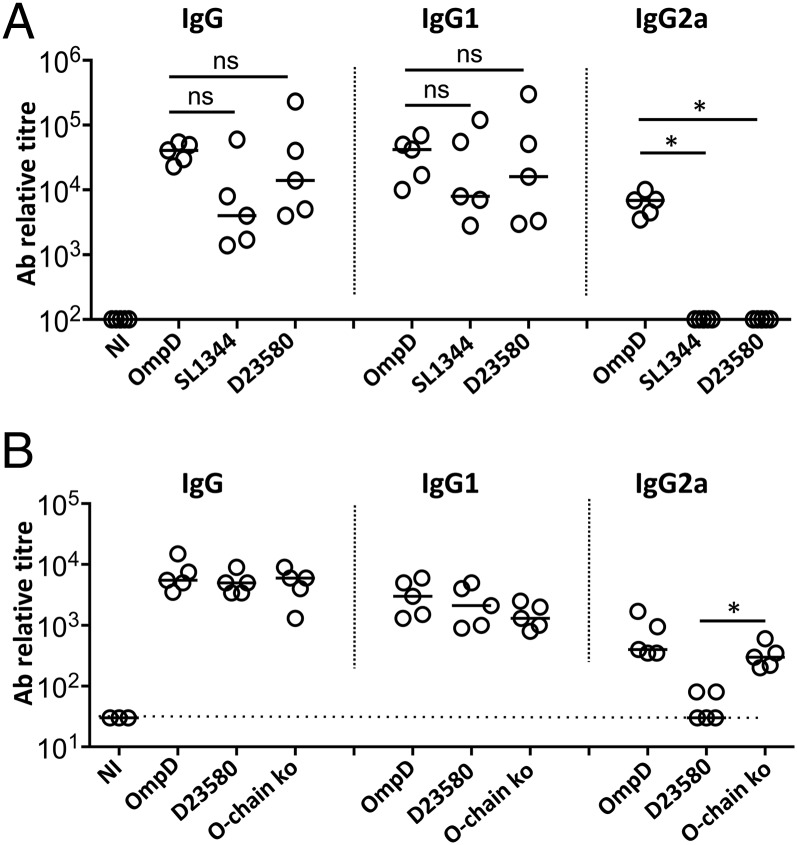
Anti-OmpD IgG2a does not bind efficiently to OmpD on the bacterial surface. WT mice were immunized with OmpD at days 0, 14, and 28. Sera were taken 7 d after the last immunization. (**A**) Anti-OmpD IgG, IgG1, or IgG2a titers were detected by ELISA using plates coupled with OmpD, *S.* Typhimurium D23580, or *S.* Typhimurium SL1344 bacteria. (**B**) Anti-OmpD IgG, IgG1, or IgG2a titers were detected by ELISA using plates coupled with OmpD, *S.* Typhimurium D23580, or *S.* Typhimurium D23580 lacking O-Ag. Data are representative of two independent experiments, and two individual replicate experiments, with at least three sera included for each group. *p < 0.05. NI, nonimmunized control serum.

The earlier experiments demonstrate that IgG1 and IgG2a, although both able to bind to purified OmpD protein (Fig. 3A), do not offer equivalent protection. One explanation for this is that the different IgG isotypes may not bind equally well to OmpD when it is found in its natural context on the bacterial surface. To test whether IgG1 and IgG2a are able to bind OmpD on the surface of intact bacteria, ELISAs were performed using free OmpD, or two different strains of intact *S.* Typhimurium bacteria (the laboratory strain SL1344 and the clinical African isolate D23580; Fig. 6A). This showed that, whereas IgG1 bound to ELISA plates coupled with intact bacteria and with purified OmpD, binding of IgG2a to intact bacteria was undetectable (Fig. 6A). To test whether the presence of LPS O-Ag may impede binding of IgG2a to OmpD, binding of total IgG, IgG1, and IgG2a to plates coated with free OmpD, whole, intact WT *S.* Typhimurium bacteria and whole, intact O-Ag–deficient *S.* Typhimurium were tested. This showed that more binding of IgG2a was detected if O-Ag was absent (Fig. 6B). Thus, OmpD-specific IgG2a is less capable of binding to WT bacteria than IgG1.

## Discussion

In this study we identify a significant role for IgG1 in the protection mediated by an experimental vaccine against nontyphoidal *Salmonella* infections. The requirement for IgG1 in such responses is surprising because infection with *S.* Typhimurium in mice primarily induces IgG2a. Experimental studies in mice suggest that after natural infection the immunodominant target of protective Ab is the serovar-specific O-Ag of LPS (30). Thus, IgG2a to LPS O-Ag may provide more effective protection than it does to OmpD. Indeed, work examining the capacity of monoclonal Abs to LPS O-Ag supports this concept, although it is of significance that monoclonal Abs that were of an IgG1 isotype also provided protection (31). Purified OmpD induces robust IgG responses of all four IgG isotypes in mice and long-lasting IgG responses in humans (32), although the isotypes induced by *Salmonella* OmpD in humans have not been assessed. The effects observed in the IgG1ko mice are unlikely to be due to non-Ab mechanisms of B cells, which can promote protection, as opsonizing bacteria with Ab prior to infection was sufficient to reproduce the defect in protection (33, 34). This supports the wealth of evidence showing an important role for Ab in protecting against *Salmonella* infections, although this protection is not complete as both T and B cell elements are required for full protection (10, 19, 21, 30, 35, 36). Our study refines our understanding of the role of IgG in protection to highlight the necessity to consider the requirement for different isotypes to different Ags in this protection.

Different IgG isotypes may make distinct contributions to protection dependent upon a number of factors, some of which are related to the intrinsic properties of the Ab isotype and some to the intrinsic nature and properties of the Ag. In humans this can be exemplified by examining the positive and negative roles of IgG2. For instance, examining the response to LPS O-Ag in human infections we have shown that the amount of Ab to O-Ag can influence the level of killing that Ab can promote (37–39). Moreover, when anti-LPS O-Ag IgG is present in supra-physiological amounts, it can inhibit bactericidal activity of Ab in cell-dependent and independent systems (38). Indeed, removing such inhibitory Abs may show clear patient benefit in some circumstances **(**39**)**. This is likely to reflect an imbalance between the amounts of Ab present and the sheer number of O-Ag epitopes found on the bacterial surface, which can run into millions. Interestingly, a single isotype, IgG2, is sufficient to account for this inhibitory effect, yet this isotype is also known to be important for protection to encapsulated bacteria and after immunization with capsular polysaccharide vaccines (37, 38, 40, 41). In addition to this there may be a role for the actual frequency that Ag is found. In contrast to the predominance of LPS molecules on the bacterial surface, OmpD is present at a much lower frequency (42) and so any excess of Abs to this Ag may be less likely to be inhibitory. Indeed, in studies examining the effect of infection with the helminth *N. brasiliensis* on immunity observed to porins, a reduced level of protection was observed associated with a reduced level of IgG1 but not IgG2a to porins after immunization (27). The importance of IgG1 seen in the current studies may therefore help explain the reduced protection induced to porins in that model.

The Th1/Th2 bias in IgG switching observed in mice is not found in humans because IgG isotypes in both species have evolved independently (43). Nevertheless, there are some parallels between individual isotypes including structural homologies between the different isotypes. This is reflected in IgG isotypes differing in their intrinsic flexibility and capacity to activate complement. Murine IgG1 and human IgG2 are predicted to be relatively rigid compared with other isotypes such as IgG2a in mice and IgG1 in humans (44). Moreover, both human IgG2 and murine IgG1 are relatively poor at activating human complement (44). Because IgG1 and IgG2a differ in their intrinsic flexibility, it is possible that this contributes to the Ab actually being able to access the epitopes on the Ag surface. To understand this requires the use of sophisticated modeling. Because anti-OmpD IgG2a showed enhanced binding to whole bacteria when O-Ag was absent, it suggests that not all epitopes proximal to the bacterial surface are accessible equally to all IgG isotypes because of occlusion by LPS O-Ag. Indeed, this is supported by studies examining the protective role of IgG Abs of multiple isotypes against LPS O-Ag (31).

This work shows the importance of inducing the appropriate isotype to achieve the maximal efficacy of a vaccine. The wider relevance of this is that the isotype induced to vaccines is more controllable and open to modulation, through use of adjuvants and the nature of the vaccine employed, than the Ab response observed after natural infection. In parallel with the increased relevance of IgG isotype in generating effective immunotherapies, it indicates that it is time to renew our focus on the attributes of different forms of IgG.

## References

[1] Bruhns P. 2012 Properties of mouse and human IgG receptors and their contribution to disease models. Blood 119: 5640–5649. 22535666 10.1182/blood-2012-01-380121

[2] Toellner K. M. Luther S. A. Sze D. M. Choy R. K. Taylor D. R. MacLennan I. C. Acha-Orbea H. 1998 T helper 1 (Th1) and Th2 characteristics start to develop during T cell priming and are associated with an immediate ability to induce immunoglobulin class switching. J. Exp. Med. 187: 1193–1204. 9547331 10.1084/jem.187.8.1193 PMC2212236

[3] Urban J. F. Jr. Noben-Trauth N. Donaldson D. D. Madden K. B. Morris S. C. Collins M. Finkelman F. D. 1998 IL-13, IL-4Ralpha, and Stat6 are required for the expulsion of the gastrointestinal nematode parasite Nippostrongylus brasiliensis. Immunity 8: 255–264. 9492006 10.1016/s1074-7613(00)80477-x

[4] Cunningham A. F. Khan M. Ball J. Toellner K. M. Serre K. Mohr E. MacLennan I. C. 2004 Responses to the soluble flagellar protein FliC are Th2, while those to FliC on *Salmonella* are Th1. Eur. J. Immunol. 34: 2986–2995. 15384042 10.1002/eji.200425403

[5] Cunningham A. F. Fallon P. G. Khan M. Vacheron S. Acha-Orbea H. MacLennan I. C. McKenzie A. N. Toellner K. M. 2002 Th2 activities induced during virgin T cell priming in the absence of IL-4, IL-13, and B cells. J. Immunol. 169: 2900–2906. 12218103 10.4049/jimmunol.169.6.2900

[6] Bournazos S. DiLillo D. J. Ravetch J. V. 2015 The role of Fc-FcγR interactions in IgG-mediated microbial neutralization. J. Exp. Med. 212: 1361–1369. 26282878 10.1084/jem.20151267 PMC4548051

[7] Barrington R. Zhang M. Fischer M. Carroll M. C. 2001 The role of complement in inflammation and adaptive immunity. Immunol. Rev. 180: 5–15. 11414363 10.1034/j.1600-065x.2001.1800101.x

[8] Yuan R. Casadevall A. Spira G. Scharff M. D. 1995 Isotype switching from IgG3 to IgG1 converts a nonprotective murine antibody to *Cryptococcus neoformans* into a protective antibody. J. Immunol. 154: 1810–1816 7836766

[9] Ravindran R. Foley J. Stoklasek T. Glimcher L. H. McSorley S. J. 2005 Expression of T-bet by CD4 T cells is essential for resistance to *Salmonella* infection. J. Immunol. 175: 4603–4610. 16177105 10.4049/jimmunol.175.7.4603

[10] Gil-Cruz C. Bobat S. Marshall J. L. Kingsley R. A. Ross E. A. Henderson I. R. Leyton D. L. Coughlan R. E. Khan M. Jensen K. T. 2009 The porin OmpD from nontyphoidal *Salmonella* is a key target for a protective B1b cell antibody response. Proc. Natl. Acad. Sci. USA 106: 9803–9808. 19487686 10.1073/pnas.0812431106 PMC2701014

[11] Secundino I. López-Macías C. Cervantes-Barragán L. Gil-Cruz C. Ríos-Sarabia N. Pastelin-Palacios R. Villasis-Keever M. A. Becker I. Puente J. L. Calva E. Isibasi A. 2006 *Salmonella* porins induce a sustained, lifelong specific bactericidal antibody memory response. Immunology 117: 59–70. 16423041 10.1111/j.1365-2567.2005.02263.x PMC1782194

[12] Lorenz M. Jung S. Radbruch A. 1995 Switch transcripts in immunoglobulin class switching. Science 267: 1825–1828. 7892607 10.1126/science.7892607

[13] Szabo S. J. Kim S. T. Costa G. L. Zhang X. Fathman C. G. Glimcher L. H. 2000 A novel transcription factor, T-bet, directs Th1 lineage commitment. Cell 100: 655–669. 10761931 10.1016/s0092-8674(00)80702-3

[14] Casola S. Cattoretti G. Uyttersprot N. Koralov S. B. Seagal J. Hao Z. Waisman A. Egert A. Ghitza D. Rajewsky K. 2006 Tracking germinal center B cells expressing germ-line immunoglobulin gamma1 transcripts by conditional gene targeting [Published erratum appears in 2007 *Proc. Natl. Acad. Sci. USA* 104: 2025.] Proc. Natl. Acad. Sci. USA 103: 7396–7401. 16651521 10.1073/pnas.0602353103 PMC1464351

[15] Hoiseth S. K. Stocker B. A. 1981 Aromatic-dependent *Salmonella typhimurium* are non-virulent and effective as live vaccines. Nature 291: 238–239. 7015147 10.1038/291238a0

[16] Flores-Langarica A. Marshall J. L. Bobat S. Mohr E. Hitchcock J. Ross E. A. Coughlan R. E. Khan M. Van Rooijen N. Henderson I. R. 2011 T-zone localized monocyte-derived dendritic cells promote Th1 priming to *Salmonella.* Eur. J. Immunol. 41: 2654–2665. 21630252 10.1002/eji.201141440

[17] Kingsley R. A. Msefula C. L. Thomson N. R. Kariuki S. Holt K. E. Gordon M. A. Harris D. Clarke L. Whitehead S. Sangal V. 2009 Epidemic multiple drug resistant *Salmonella* Typhimurium causing invasive disease in sub-Saharan Africa have a distinct genotype. Genome Res. 19: 2279–2287. 19901036 10.1101/gr.091017.109 PMC2792184

[18] Datsenko K. A. Wanner B. L. 2000 One-step inactivation of chromosomal genes in *Escherichia coli* K-12 using PCR products. Proc. Natl. Acad. Sci. USA 97: 6640–6645. 10829079 10.1073/pnas.120163297 PMC18686

[19] Cunningham A. F. Gaspal F. Serre K. Mohr E. Henderson I. R. Scott-Tucker A. Kenny S. M. Khan M. Toellner K. M. Lane P. J. MacLennan I. C. 2007 *Salmonella* induces a switched antibody response without germinal centers that impedes the extracellular spread of infection. J. Immunol. 178: 6200–6207. 17475847 10.4049/jimmunol.178.10.6200

[20] Salazar-González R. M. Maldonado-Bernal C. Ramírez-Cruz N. E. Rios-Sarabia N. Beltrán-Nava J. Castañón-González J. Castillo-Torres N. Palma-Aguirre J. A. Carrera-Camargo M. López-Macías C. Isibasi A. 2004 Induction of cellular immune response and anti-*Salmonella enterica* serovar typhi bactericidal antibodies in healthy volunteers by immunization with a vaccine candidate against typhoid fever. Immunol. Lett. 93: 115–122. 15158606 10.1016/j.imlet.2004.01.010

[21] Marshall J. L. Flores-Langarica A. Kingsley R. A. Hitchcock J. R. Ross E. A. López-Macías C. Lakey J. Martin L. B. Toellner K. M. MacLennan C. A. 2012 The capsular polysaccharide Vi from *Salmonella typhi* is a B1b antigen. J. Immunol. 189: 5527–5532. 23162127 10.4049/jimmunol.1103166 PMC3605773

[22] Toellner K. M. Gulbranson-Judge A. Taylor D. R. Sze D. M. MacLennan I. C. 1996 Immunoglobulin switch transcript production in vivo related to the site and time of antigen-specific B cell activation. J. Exp. Med. 183: 2303–2312. 8642339 10.1084/jem.183.5.2303 PMC2192570

[23] Zhang Y. Meyer-Hermann M. George L. A. Figge M. T. Khan M. Goodall M. Young S. P. Reynolds A. Falciani F. Waisman A. 2013 Germinal center B cells govern their own fate via antibody feedback. J. Exp. Med. 210: 457–464. 23420879 10.1084/jem.20120150 PMC3600904

[24] Ralph P. Prichard J. Cohn M. 1975 Reticulum cell sarcoma: an effector cell in antibody-dependent cell-mediated immunity. J. Immunol. 114: 898–905 1089721

[25] Voelz K. Lammas D. A. May R. C. 2009 Cytokine signaling regulates the outcome of intracellular macrophage parasitism by *Cryptococcus neoformans* [Published erratum appears in 2016 *Infect. Immun.* 84: 3656.] Infect. Immun. 77: 3450–3457. 19487474 10.1128/IAI.00297-09 PMC2715691

[26] Abramoff M. D. Magelhaes P. J. Ram S. J. 2004 Image processing with ImageJ. Biophoton. Int. 11: 36–42.

[27] Bobat S. Darby M. Mrdjen D. Cook C. Logan E. Auret J. Jones E. Schnoeller C. Flores-Langarica A. Ross E. A. 2014 Natural and vaccine-mediated immunity to *Salmonella* Typhimurium is impaired by the helminth *Nippostrongylus brasiliensis.* PLoS Negl. Trop. Dis. 8: e3341. 25474738 10.1371/journal.pntd.0003341 PMC4256288

[28] Uppington H. Menager N. Boross P. Wood J. Sheppard M. Verbeek S. Mastroeni P. 2006 Effect of immune serum and role of individual Fcgamma receptors on the intracellular distribution and survival of *Salmonella enterica* serovar Typhimurium in murine macrophages. Immunology 119: 147–158. 16836651 10.1111/j.1365-2567.2006.02416.x PMC1782356

[29] Gondwe E. N. Molyneux M. E. Goodall M. Graham S. M. Mastroeni P. Drayson M. T. MacLennan C. A. 2010 Importance of antibody and complement for oxidative burst and killing of invasive nontyphoidal *Salmonella* by blood cells in Africans. Proc. Natl. Acad. Sci. USA 107: 3070–3075. 20133627 10.1073/pnas.0910497107 PMC2840319

[30] Hormaeche C. E. Mastroeni P. Harrison J. A. Demarco de Hormaeche R. Svenson S. Stocker B. A. 1996 Protection against oral challenge three months after i.v. immunization of BALB/c mice with live Aro *Salmonella typhimurium* and *Salmonella enteritidis* vaccines is serotype (species)-dependent and only partially determined by the main LPS O antigen. Vaccine 14: 251–259. 8744548 10.1016/0264-410x(95)00249-z

[31] Goh Y. S. Clare S. Micoli F. Saul A. Mastroeni P. MacLennan C. A. 2015 Monoclonal antibodies of a diverse isotype induced by an O-antigen glycoconjugate vaccine mediate in vitro and in vivo killing of African invasive nontyphoidal *Salmonella.* Infect. Immun. 83: 3722–3731. 26169269 10.1128/IAI.00547-15 PMC4534659

[32] Perez-Shibayama C. Gil-Cruz C. Pastelin-Palacios R. Cervantes-Barragan L. Hisaki E. Chai Q. Onder L. Scandella E. Regen T. Waisman A. 2014 IFN-γ-producing CD4+ T cells promote generation of protective germinal center-derived IgM+ B cell memory against Salmonella Typhi. J. Immunol. 192: 5192–5200. 24778443 10.4049/jimmunol.1302526

[33] Nanton M. R. Way S. S. Shlomchik M. J. McSorley S. J. 2012 Cutting edge: B cells are essential for protective immunity against *Salmonella* independent of antibody secretion. J. Immunol. 189: 5503–5507. 23150714 10.4049/jimmunol.1201413 PMC3518619

[34] Horsnell W. G. Darby M. G. Hoving J. C. Nieuwenhuizen N. McSorley H. J. Ndlovu H. Bobat S. Kimberg M. Kirstein F. Cutler A. J. 2013 IL-4Rα-associated antigen processing by B cells promotes immunity in Nippostrongylus brasiliensis infection. PLoS Pathog. 9: e1003662. 24204255 10.1371/journal.ppat.1003662 PMC3812011

[35] McSorley S. J. Jenkins M. K. 2000 Antibody is required for protection against virulent but not attenuated *Salmonella enterica* serovar typhimurium. Infect. Immun. 68: 3344–3348. 10816483 10.1128/iai.68.6.3344-3348.2000 PMC97596

[36] Mastroeni P. Villarreal-Ramos B. Hormaeche C. E. 1993 Adoptive transfer of immunity to oral challenge with virulent salmonellae in innately susceptible BALB/c mice requires both immune serum and T cells. Infect. Immun. 61: 3981–3984. 8359920 10.1128/iai.61.9.3981-3984.1993 PMC281103

[37] MacLennan C. A. Gilchrist J. J. Gordon M. A. Cunningham A. F. Cobbold M. Goodall M. Kingsley R. A. van Oosterhout J. J. Msefula C. L. Mandala W. L. 2010 Dysregulated humoral immunity to nontyphoidal *Salmonella* in HIV-infected African adults. Science 328: 508–512. 20413503 10.1126/science.1180346 PMC3772309

[38] Wells T. J. Whitters D. Sevastsyanovich Y. R. Heath J. N. Pravin J. Goodall M. Browning D. F. O’Shea M. K. Cranston A. De Soyza A. 2014 Increased severity of respiratory infections associated with elevated anti-LPS IgG2 which inhibits serum bactericidal killing. J. Exp. Med. 211: 1893–1904. 25113975 10.1084/jem.20132444 PMC4144740

[39] Wells T. J. Davison J. Sheehan E. Kanagasundaram S. Spickett G. MacLennan C. A. Stockley R. A. Cunningham A. F. Henderson I. R. De Soyza A. 2017 The use of plasmapheresis in patients with bronchiectasis with *Pseudomonas aeruginosa* infection and inhibitory antibodies. Am. J. Respir. Crit. Care Med. 195: 955–958. 28362198 10.1164/rccm.201603-0599LE

[40] Lane P. J. MacLennan I. C. 1986 Impaired IgG2 anti-pneumococcal antibody responses in patients with recurrent infection and normal IgG2 levels but no IgA. Clin. Exp. Immunol. 65: 427–433. 3791703 PMC1542305

[41] Goh Y. S. Necchi F. O’Shaughnessy C. M. Micoli F. Gavini M. Young S. P. Msefula C. L. Gondwe E. N. Mandala W. L. Gordon M. A. 2016 Bactericidal immunity to *Salmonella* in Africans and mechanisms causing its failure in HIV infection. PLoS Negl. Trop. Dis. 10: e0004604. 27057743 10.1371/journal.pntd.0004604 PMC4825999

[42] Nikaido H. 2003 Molecular basis of bacterial outer membrane permeability revisited. Microbiol. Mol. Biol. Rev. 67: 593–656. 14665678 10.1128/MMBR.67.4.593-656.2003 PMC309051

[43] Hayashida H. Miyata T. Yamawaki-Kataoka Y. Honjo T. Wels J. Blattner F. 1984 Concerted evolution of the mouse immunoglobulin gamma chain genes. EMBO J. 3: 2047–2053. 6436019 10.1002/j.1460-2075.1984.tb02090.x PMC557642

[44] Dangl J. L. Wensel T. G. Morrison S. L. Stryer L. Herzenberg L. A. Oi V. T. 1988 Segmental flexibility and complement fixation of genetically engineered chimeric human, rabbit and mouse antibodies. EMBO J. 7: 1989–1994. 3138110 10.1002/j.1460-2075.1988.tb03037.x PMC454472

